# Role of gut microbiota metabolites against vein graft restenosis: insights from network pharmacology, molecular docking and molecular dynamic simulation

**DOI:** 10.1186/s12920-025-02290-6

**Published:** 2025-12-08

**Authors:** Zi’ang Li, Xiankun Liu, Yiming Bai, Yunpeng Bai, Zhigang Guo

**Affiliations:** 1 Clinical School of Thoracic, Tianjin Medical University, Tianjin, 300222 China; 2 Department of Cardiac Surgery, Tianjin Chest Hospital, Tianjin Medical University, Tianjin, 300222 China; 3 Department of Cardiac Surgery, The First Affiliated Hospital of Shandong First Medical University & Shandong Provincial Qianfoshan Hospital, Jinan, 250000 China

**Keywords:** Vein graft restenosis (VGR), Gut microbiota, Metabolites, Network pharmacology

## Abstract

**Background:**

Gut microbiota metabolites are increasingly recognized for their role in modulating chronic disease progression. However, their potential impact on vein graft restenosis (VGR) remains unexplored. This study aimed to elucidate the mechanisms by which gut microbiota and its metabolites attenuate VGR using an integrated approach of network pharmacology, molecular docking, and molecular dynamics (MD) simulations.

**Methods:**

Gut microbiota, metabolites, and human gut targets were obtained from the gutMGene database. Metabolite targets were predicted using SwissTargetPrediction and Similarity Ensemble Approach, while disease targets were collected from GeneCards, Online Mendelian Inheritance in Man (OMIM), and DrugBank. Overlapping targets were used to construct both a protein-protein interaction (PPI) network and a gut microbiota–metabolites–targets–VGR (GM-M-T-V) network to identify key microbiota, core metabolites, and hub targets. Enrichment analysis investigated associated biological processes, cellular components, molecular functions, and signaling pathways. Drug-likeness and toxicity were evaluated with SwissADME and ADMETlab 2.0. Molecular docking and MD simulations assessed the binding affinity and dynamic characteristics of target-metabolite complexes.

**Results:**

Integrated data from relevant databases identified 260 gut microbiota, 251 metabolites, 404 metabolite targets, 238 human gut targets, and 741 VGR-related targets. Among these, 16 overlapping targets were identified for further analysis. Enrichment analysis highlighted significant involvement of the relaxin signaling pathway, while PPI topology analysis pinpointed AKT1, NFKB1, EGFR, PTGS2, and PPARG as hub targets. Quercetin was prioritized as the core metabolite based on its top network connectivity, favorable drug-likeness prediction, and manageable in silico-predicted hepatotoxicity/genotoxicity risks in light of its absent clinical toxicity. Molecular docking revealed that quercetin bound to four hub targets (AKT1, NFKB1, EGFR, PPARG) with affinities (ranging from−6.0 to−8.9 kcal/mol) comparable or superior to positive controls. MD simulations further suggested favorable structural stability and binding affinity of the EGFR–quercetin complex.

**Conclusion:**

This integrative study elucidates the role of gut microbiota metabolites against VGR, identifying the microbial metabolite quercetin as a promising multi-target therapeutic agent primarily via the relaxin signaling pathway, which provides a mechanistic groundwork for a novel potential treatment strategy.

**Supplementary Information:**

The online version contains supplementary material available at 10.1186/s12920-025-02290-6.

## Introduction

 Coronary artery bypass grafting (CABG) remains a cornerstone in the treatment of coronary heart disease (CHD). However, post-surgical vein graft restenosis (VGR) remains a major clinical challenge. The primary pathological mechanism driving VGR is intimal hyperplasia, which is largely triggered by inflammation and oxidative stress. Specifically, surgical vascular injury causes endothelial cell damage and apoptosis. This in turn promotes the proliferation and migration of vascular smooth muscle cells (VSMCs) toward the site of injury, ultimately leading to intimal hyperplasia [1]. Furthermore, the vein graft exposed to the arterial circulation experiences a marked increase in hemodynamic pressure and shear stress, which further stimulates VSMC proliferation and migration, thereby contributing to restenosis [2]. VGR not only impairs patients’ quality of life but also raises the risk of serious cardiovascular events, adding substantially to the healthcare burden. Therefore, developing integrated strategies to effectively inhibit VGR is critically needed.

In recent years, gut microbiota metabolites have become a central focus in medical research. Groundbreaking studies have identified the gut microbial metabolite imidazole propionate (ImP) as an independent promoter of atherosclerosis, underscoring the therapeutic potential of targeting receptors for specific microbiota-derived metabolites [3, 4]. The gut microbiota itself is dominated by two primary phyla, *Firmicutes* and *Bacteroidetes*, which are essential for maintaining intestinal functionality [5]. Through metabolites such as short-chain fatty acids (SCFAs), amino acids, and bile acids, the gut microbiota profoundly influences host metabolism and immune function [6, 7]. These metabolites can indirectly affect cardiovascular health by modulating systemic inflammation, metabolic pathways, and myocardial performance [8–10]. The mechanisms of gut microbiota metabolites are hypothesized to involve two primary modes of action: a systemic mode, where metabolites enter the circulation and interact directly with vascular targets akin to single-component drugs, and a local mode, where they bind to intestinal targets and remotely modulate vascular health via the gut-vascular axis—a mechanism supported by a growing body of hypothesis-driven research.

The composition of the gut microbiota is not static but undergoes dynamic shifts in response to environmental, dietary, and health-related factors. These shifts are increasingly linked to cardiovascular disease pathogenesis. Studies have demonstrated that an altered gut microbiota profile, characterized by reduced microbial diversity and an elevated *Firmicutes*/*Bacteroidetes* ratio, is associated with hypertension [11]. Mechanistically, SCFAs produced by gut bacteria can regulate macrophage activity, thereby curbing the release of pro-inflammatory factors and mitigating the chronic inflammation that drives atherosclerotic plaque formation [12, 13]. Furthermore, SCFAs are recognized for their direct cardioprotective benefits, which include improving cardiac output and attenuating heart failure progression [14]. Consequently, the gut microbiota and its metabolites exert systemic physiological influences that extend well beyond digestive health. They regulate host metabolism and immunity via multiple interconnected pathways, positioning them as potential key contributors to cardiovascular disease pathogenesis. Given this, the pharmacological repurposing of gut microbiota metabolites represents a promising therapeutic strategy for mitigating VGR, although the precise underlying mechanisms require further elucidation.

**Fig. 1:**
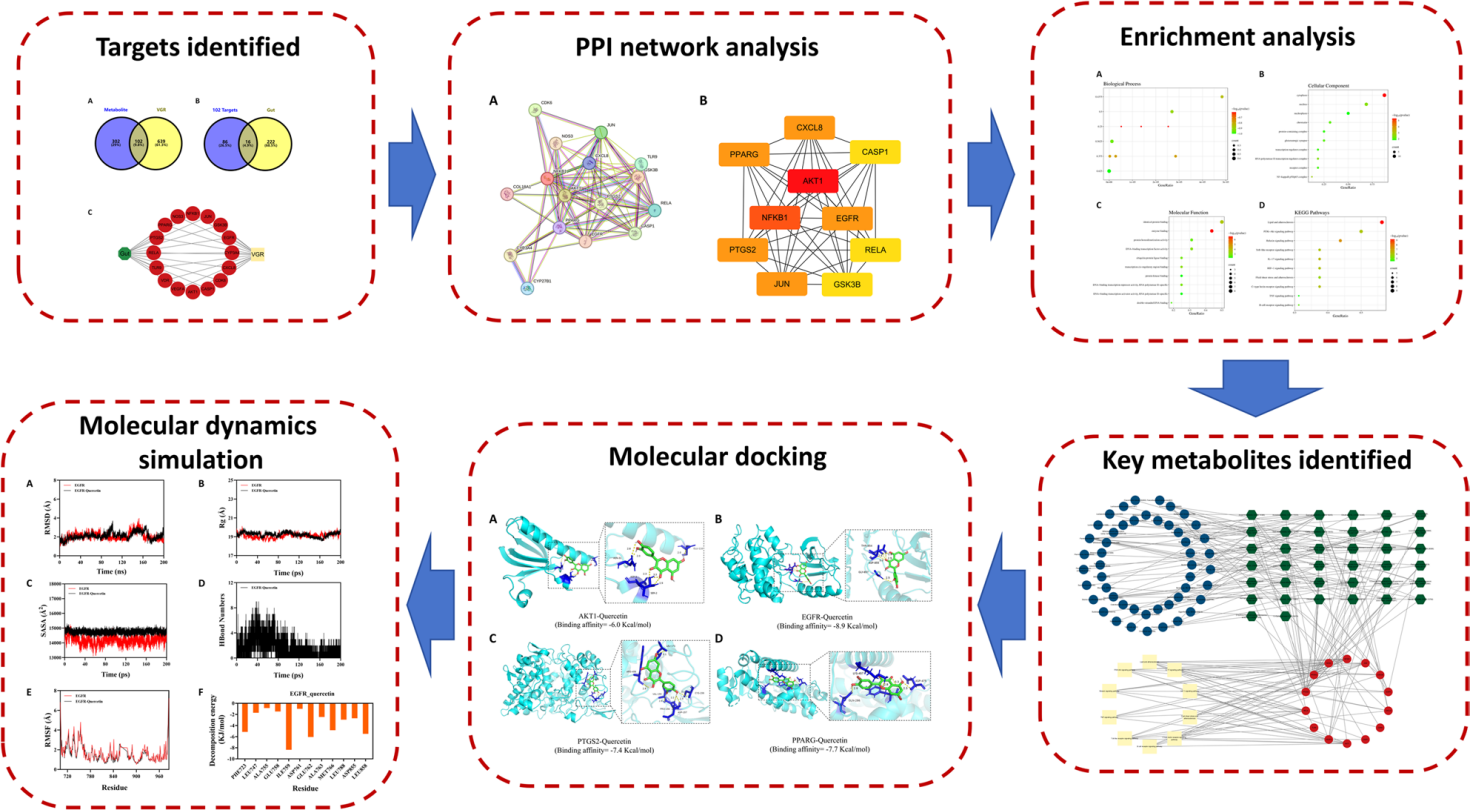
The flowchart of this research

This study employed network pharmacology, molecular docking, and molecular dynamics (MD) simulations to systematically analyze the role of gut microbiota metabolites on VGR, emphasizing on elucidating the interaction mechanisms between metabolites and targets (Fig. 1). By integrating multiple data sources, this research investigated the potential effectiveness of gut microbiota metabolites, with the objective of offering innovative insights and treatment strategies for preventing VGR.

## Methods and materials

### Identification of gut microbiota metabolites and potential targets

Corresponding relationships between gut microbiota and its metabolites was sourced from the gutMGene database (http://bio-computing.hrbmu.edu.cn/gutmgene/#/home) [15]. The canonical SMILES of the metabolites were obtained from the PubChem database (https://pubchem.ncbi.nlm.nih.gov/) using the corresponding PubChem CID provided by the gutMGene database [16]. Specifically, only the canonical SMILES notation was collected for each metabolite to ensure a consistent structural representation and compatibility with the diverse bioinformatics tools in our analysis pipeline. However, we acknowledge that this approach does not capture specific stereochemical information, which represents a limitation for interpreting the bioactivity of stereospecific metabolites. Subsequently, the Canonical SMILES of gut microbiota metabolites were submitted to SwissTargetPrediction (http://www.swisstargetprediction.ch/) and Similarity Ensemble Approach (https://sea.bkslab.org/) for predicting potential targets [17, 18]. To improve the accuracy of target prediction, Venny 2.1.0 (https://bioinfogp.cnb.csic.es/tools/venny/index.html) was employed to identify the intersection of predicted targets. Metabolites that lack overlapping targets were excluded. All databases and servers mentioned above were accessed in March 2025.

### Identification of disease targets and human gut targets

The disease targets were sourced from GeneCards database (http://www.genecards.org/), Online Mendelian Inheritance in Man (OMIM, https://omim.org/), and DrugBank (https://go.drugbank.com/) using the keyword “vein graft restenosis” [19–21]. The human gut targets were obtained from the gutMGene database. Subsequently, Venny 2.1.0 was utilized to generate the Venn diagram in order to identify and illustrate the overlapping targets among metabolites, VGR, and human gut targets. All databases and servers mentioned above were accessed in March 2025.

### Construction of protein-protein interaction network

The overlapping targets were submitted to the STRING database (https://string-db.org/) for the purpose of constructing a protein-protein interaction (PPI) network (accessed in March 2025) [22]. The species was limited to “Homo sapiens,” and the threshold was set to “medium confidence >0.4.” Subsequently, analysis and visualization of the PPI network were performed using Cytoscape v3.9.1 (www.cytoscape.org/).

In the PPI network analysis, the cytoHubba plugin (version 0.1) was utilized to identify hub targets based on 12 algorithms. To comprehensively evaluate the topological importance of nodes, we employed a variety of centrality metrics and categorized them into four classes: (1) Adjacency-based metrics, including Degree (the number of direct neighbor connections) and Maximum Neighborhood Component (MNC, equivalent to Degree); (2) Shortest path-based metrics, including Betweenness (the frequency of lying on the shortest paths between all node pairs), Closeness (the reciprocal of the average shortest path distance to all other nodes), Eccentricity (the longest shortest path distance from the node to any other node), Radiality (measuring how close a node is to the center of the network), BottleNeck (a metric identifying nodes that act as critical gateways for information flow by lying on the highest proportion of shortest paths), and Stress (the absolute number of shortest paths passing through the node); (3) Network aggregation structure-based metrics, including Maximal Clique Centrality (MCC, the number of maximal cliques a node participates in), Density of Maximum Neighborhood Component (DMNC, the connection tightness between a node and its direct neighbors), and Clustering Coefficient (the likelihood that the neighbors of a node are connected to each other); (4) Network robustness-based metrics, including Edge Percolated Component (EPC, the probability of a node remaining in the largest connected component after simulations of random edge removal). In the PPI network, nodes that were ranked within the top 5 by at least 6 different algorithms were defined as hub targets.

### Gene ontology function analysis and Kyoto encyclopaedia of genes and genomes pathway analysis

The overlapping targets were submitted to the DAVID database (https://david.ncifcrf.gov/) for “Homo sapiens” with a significance threshold of “P < 0.05” (accessed in March 2025) [23]. Gene Ontology (GO) enrichment analysis was conducted to study biological processes (BP), cellular components (CC), and molecular functions (MF). Kyoto Encyclopedia of Genes and Genomes (KEGG) enrichment analysis was performed to investigate the relevant signaling pathways. Subsequently, bubble charts were generated using the online bioinformatics drawing platform (www.bioinformatics.com.cn/) to illustrate the top 10 enriched items identified in GO and KEGG analyses (accessed in March 2025).

### Construction of the gut microbiota-metabolites-targets-signaling pathways network

Following the identification of overlapping targets, a series of networks were constructed in Cytoscape (v3.9.1) to map the interrelationships among gut microbiota, metabolites, targets, and signaling pathways. Topological analysis within the same environment identified the top 5 essential metabolites using the cytoHubba plugin (v0.1), applying Degree as the central metric—where higher values indicate greater functional importance. The resulting network architecture further enabled systematic tracing of core metabolites: backward to their microbial sources and forward to their downstream targets. Finally, the drug-likeness and toxicity evaluation of these candidate metabolites were computationally evaluated.

### Evaluation of drug-likeness and toxicity

SwissADME (http://www.swissadme.ch/index.php) was used to evaluate the drug-likeness of metabolites [24]. Lipinski’s Rule of Five was applied to evaluate the pharmacological activity of these metabolites. The evaluation criteria of drug-likeness included molecular weight (MW) ≤ 500 g/mol, H-bond acceptors (HBA) ≤ 10, H-bond donors (HBD) ≤ 5, and Consensus Log Po/w ≤ 5. Subsequently, ADMETlab 2.0 (https://admetmesh.scbdd.com/service/evaluation/cal) was utilized to evaluate the toxicity of metabolites [25]. The evaluation indicators of toxicity included hERG blockers, human hepatotoxicity (H-HT), drug-induced liver injury (DILI), AMES toxicity, rat oral acute toxicity, food and drug administration microbial data deviation investigation (FDAMDD), carcinogenicity, and respiratory toxicity. While ADMETlab 2.0 provides valuable toxicity predictions through its machine and deep learning algorithms, its computational nature necessitates experimental verification to ensure biological relevance. Therefore, following the in silico assessment, we systematically compared the predictions with experimentally validated records from the PubChem database and/or LiverTox database (https://www.ncbi.nlm.nih.gov/books/NBK547852/*)* to enhance the practical reliability of our toxicity evaluation. Based on the evaluation of drug-likeness and toxicity, combined with network Degree values, we identified the most promising metabolite for subsequent computational simulation. All databases and servers mentioned above were accessed in March 2025.

### Computational molecular docking

To assess the binding interactions between protein targets and ligand, molecular docking was performed using Autodock Vina 1.2.2. To ensure the reliability of the docking parameters and protocol, a relative efficiency evaluation was first conducted. For each target protein, if the crystal structure contained a co-crystallized ligand, a re-docking procedure was performed. Specifically, the native ligand was extracted from the protein-ligand complex and subsequently re-docked into the original active site using identical grid parameters and docking program. The root mean square deviation (RMSD) between the docked conformation and the original crystallographic conformation was calculated to assess the accuracy of the docking protocol. An RMSD value of less than 2.0 Å is generally considered acceptable, indicating a reliable setup. Furthermore, for each target, at least one known active inhibitor, either clinical or reported in the literature, was also docked as a positive control to provide a reference benchmark for the binding affinity of quercetin.

The 3D structures of all ligands were retrieved from the PubChem database and preprocessed for energy minimization using Chem3D. Protein structures were retrieved from the Protein Data Bank (PDB, https://www.rcsb.org/) [26]. The selection was based on a multi-criteria hierarchy to ensure high fidelity and biological relevance. Structures were prioritized first by resolution (<2.5 Å)and the free R-factor (R-free<0.30) to guarantee model reliability. Subsequently, the completeness of the sequence in regions of interset and the presence of relevant biological assemblies or co-crystallized ligands were critically assessed to confirm functional context. All databases mentioned above were accessed in March 2025. Proteins were prepared by removing redundant components and water molecules, repairing missing residues, assigning protonation states, adding hydrogen atoms, assigning partial charges, and optimizing side-chain conformations using Pymol 2.6.0, PDB2PQR, and AutoDock Tools. The resulting structures were energy-minimized to ensure structural stability prior to docking. For each protein target (AKT1: 1UNQ, NFKB1: 8TQD, EGFR: 8A27, PTGS2: 5F19, PPARG: 8BF1), the grid box was centered on the binding site of the target protein. A consistent grid box dimension of 25 × 25 × 25 Å was applied to all target proteins during molecular docking. Using Autodock Vina 1.2.2, global docking was performed to assess the binding affinity and characterize the binding mode between the ligands and protein targets. The binding affinity was assessed based on the calculated binding energy, and the specific binding mode was analyzed using Pymol 2.6.0 to elucidate key molecular interactions.

### Molecular dynamics simulations

To evaluate the binding stability and dynamic behavior of the EGFR-quercetin complex in a physiological environment, molecular dynamics simulations using GROMACS−2022 were performed. The protein was parameterized utilizing the Amber14SB force field, while the topology parameters for quercetin were generated using the General AMBER Force Field 2 (GAFF2). The docked EGFR-quercetin complex was solvated under Periodic Boundary Conditions (PBC) in a cubic water box. The minimum distance between the box boundary and the protein complex was set to 1.0 nm to prevent any self-interaction of the protein during simulation, resulting in a final box size of approximately 100 × 100 × 100 Å. The TIP3P water model was employed to fill the box with water molecules. The system was neutralized with ions using the GROMACS gmx genion tool, followed by energy minimization employing the steepest descent algorithm for 3,000 steps and subsequently the conjugate gradient method for an additional 2,000 steps.

Following energy minimization, the system underwent a two-step equilibration process. First, an NVT equilibration (constant number of particles, volume, and temperature) was applied for 500 ps to maintain a temperature of 310 K. This was immediately followed by an NPT equilibration (constant number of particles, pressure, and temperature) for 100 ps to maintain a pressure at 1 bar. The final production simulations was performed under the NPT ensemble for a duration of 200 ns. Temperature was maintained at 310 K, and pressure was maintained at 1 bar. All bonds were constrained using the SHAKE algorithm, allowing for an integration time step of 1.0 fs. The Particle Mesh Ewald (PME) method was employed for long-range electrostatic interactions. The Verlet cutoff scheme was used, with a cutoff at 1.0 nm for both Van der Waals and short-range electrostatic interactions. Trajectory coordinates were saved every 10 ps for subsequent analysis. The entire solvated system comprised approximately 79,540 atoms. After simulation, the trajectories were analyzed for the following metrics using built-in GROMACS tools: root mean square deviation (RMSD), radius of gyration (Rg), solvent accessible surface area (SASA), hydrogen bonds, root mean square fluctuation (RMSF), and binding free energy (using MM/PBSA approach).

## Results

### Identifying targets of gut microbiota metabolites that inhibit VGR

**Fig. 2:**
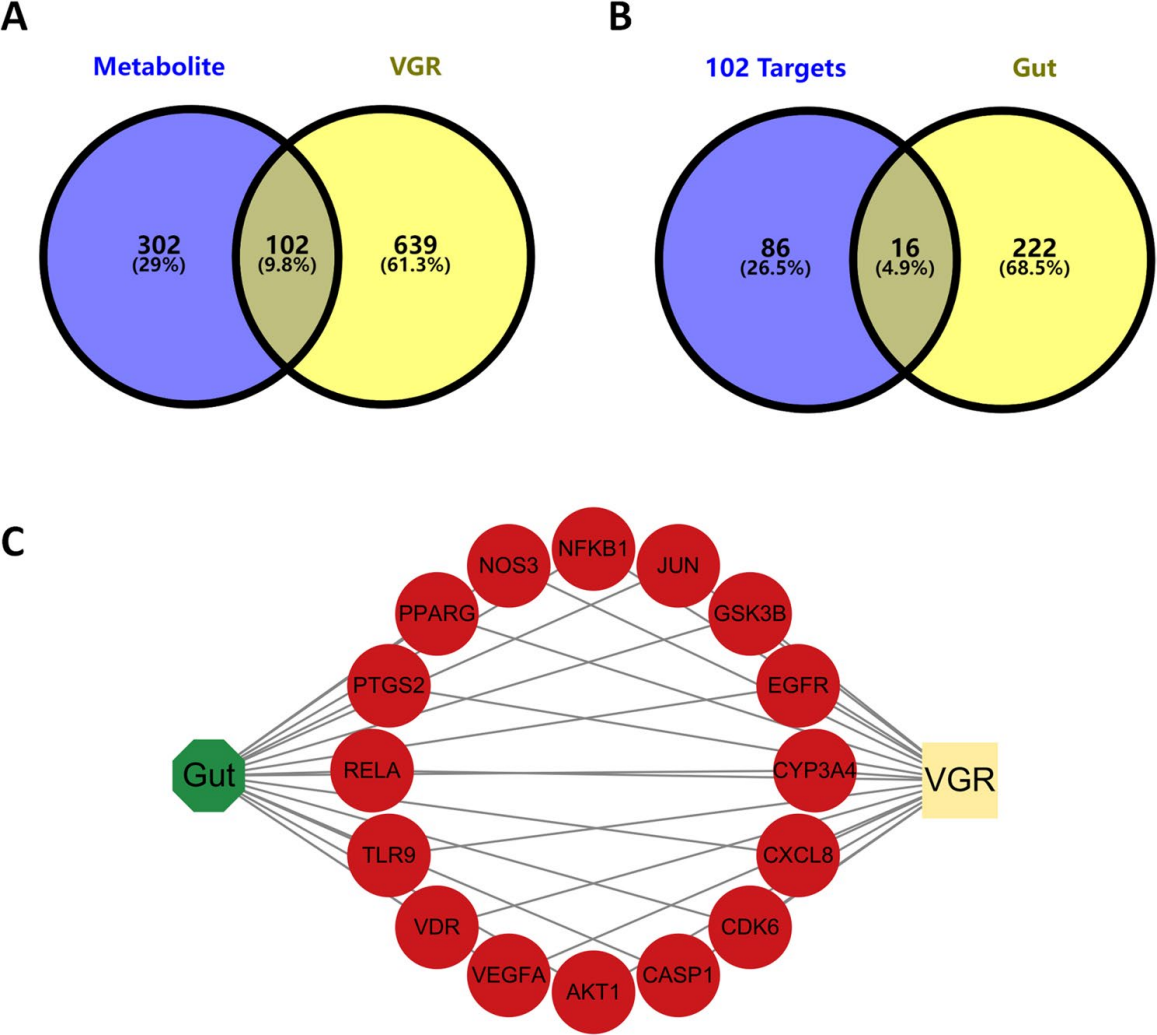
Identification of therapeutic targets. (**A**) The overlapping targets between gut microbiota metabolites and VGR; (**B**) The overlapping targets between metabolites-VGR and human gut targets; (**C**) The gut-targets-VGR network

**Fig. 3:**
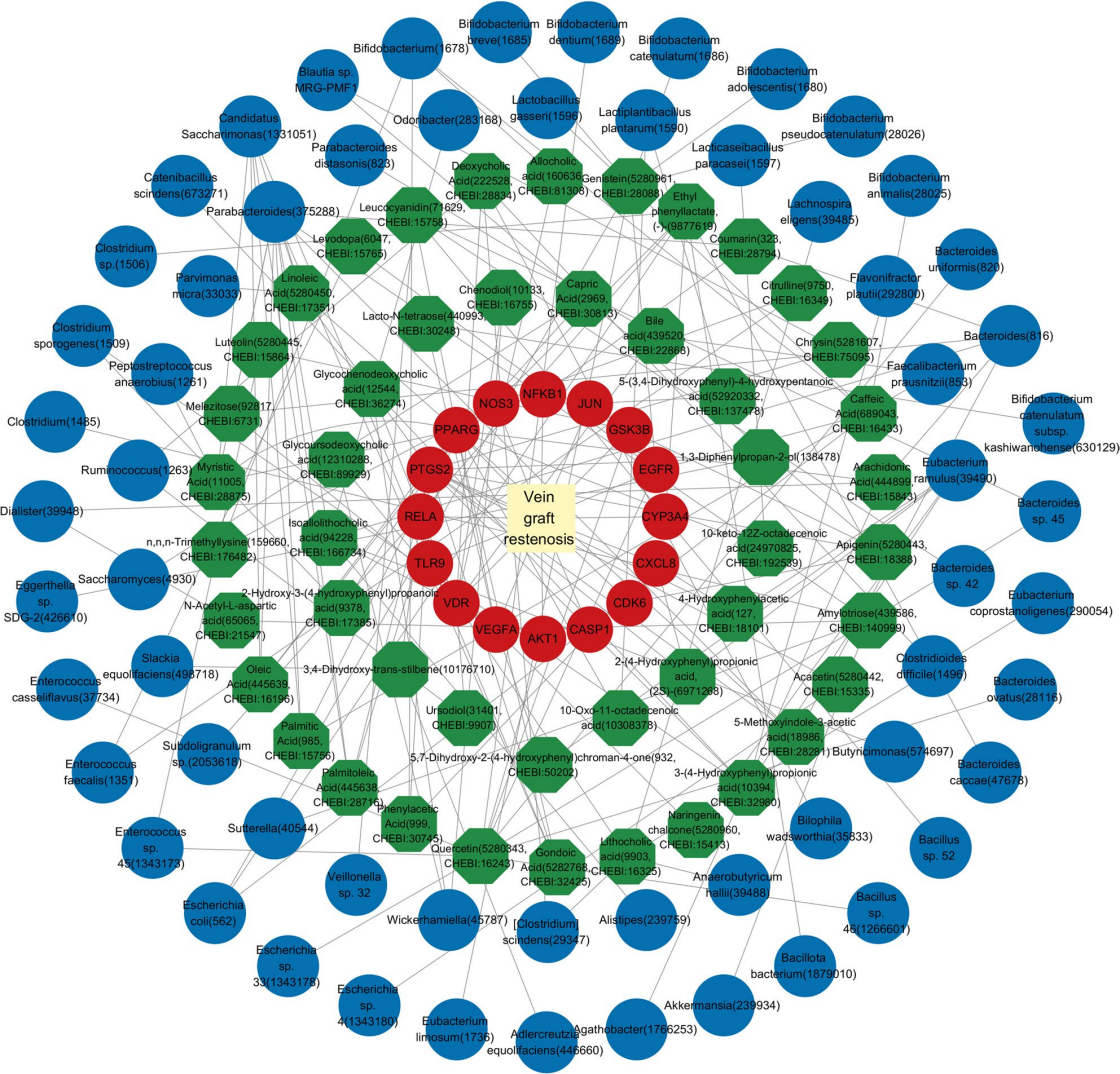
The network of gut microbiota-metabolites-targets-VGR (GM-M-T-V). In this network, the gut microbiota were depicted in blue, metabolites were represented in green, targets were illustrated in red, and VGR was highlighted in yellow

We retrieved associations between 260 gut microbiota and 251 metabolites from the gutMGene database(Additional fle 1: Table S1). Canonical SMILES of metabolites were submitted to STP and SEA for the purpose of target prediction. Those metabolites that were unavailable or lacked canonical SMILES in PubChem were excluded. Using Venn 2.1.0, a total of 404 predicted targets were identified among 156 gut microbiota metabolites (Fig. 2A). Based on the gutMGene database and predicted targets, we established correlations among gut microbiota, metabolites, and targets. The GeneCards, OMIM, and DrugBank databases collectively identified 741 disease targets. A total of 238 human gut targets were obtained from the gutMGene database. Subsequently, the Venn diagram indicated that there are 16 overlapping targets among the metabolites, VGR, and human gut targets (Fig. 2B). These overlapping targets included the following: RELA, PPARG, VEGFA, VDR, CYP3A4, PTGS2, EGFR, GSK3B, AKT1, NFKB1, NOS3, CXCL8, CDK6, CASP1, TLR9 and JUN (Fig. 2C). By reverse screening the overlapping targets, we identified 47 corresponding metabolites and 61 gut microbiota༈Additional fle 2: Table S2༉. Subsequently, we constructed a gut microbiota–metabolites–targets–VGR (GM-M-T-V) network (Fig. 3), which comprises 61 gut microbiota, 47 metabolites, and 16 overlapping targets.

### Identification of hub targets through PPI network

**Fig. 4:**
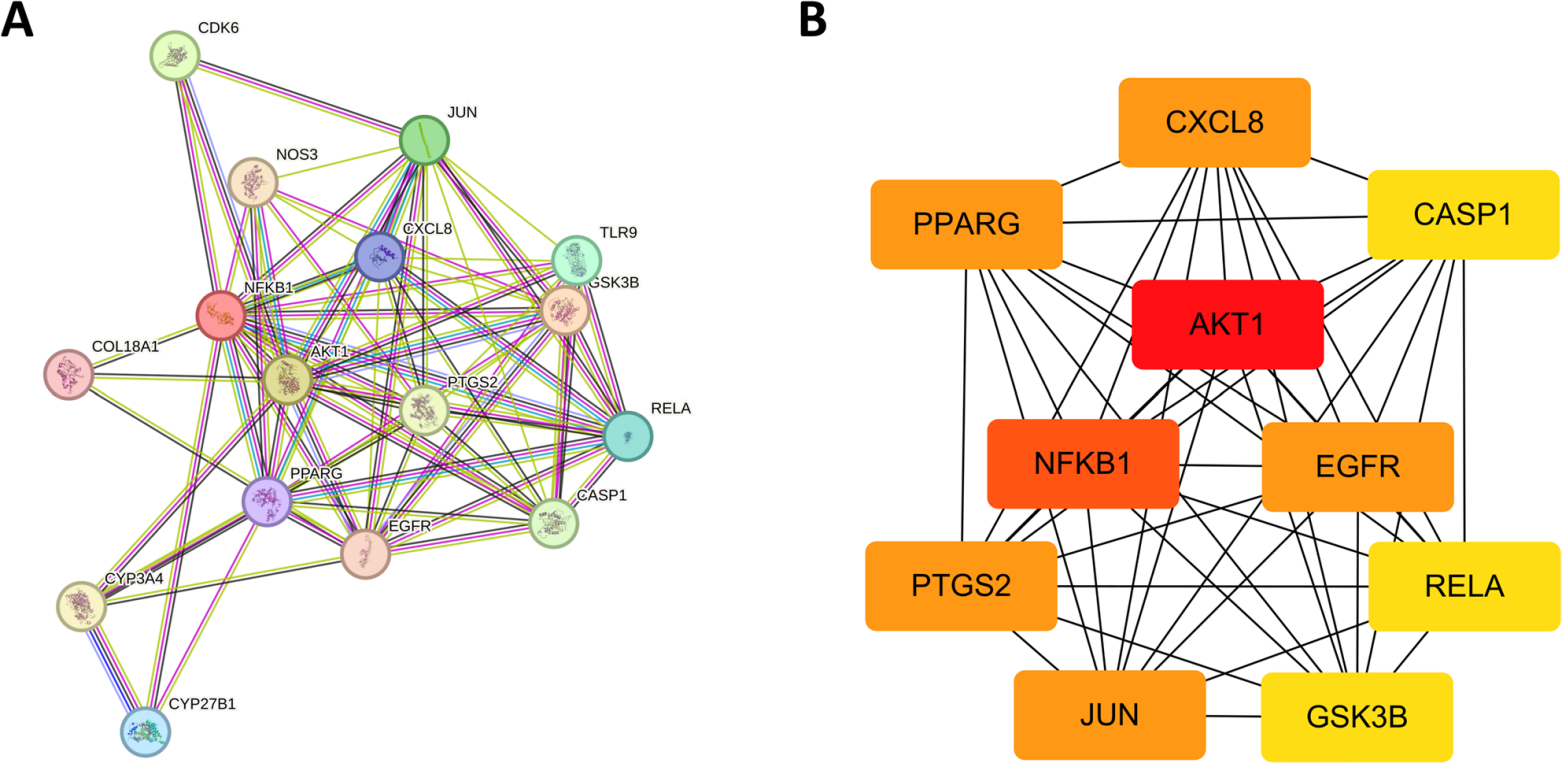
Identification of hub targets. (**A**) PPI network; (**B**) Top 10 hub targets

The overlapping targets were submitted to the STRING database to establish the PPI network (Fig. 4A), which contained 16 nodes and 73 edges, capturing both the direct and indirect regulatory roles of these targets. Subsequently, the hub targets were identified based on 12 algorithms (Table 1). The visualization of the top 10 targets based on degree value was presented in Fig. 4B. AKT1, NFKB1, and EGFR were consistently ranked among the top 5 nodes across eight algorithms, while PTGS2 and PPARG were ranked within the top 5 in seven algorithms, leading to their identification as hub targets (Table 2). The hub targets identified based on degree value also included AKT1, NFKB1, EGFR, PTGS2, and PPARG.

**Table 1:** Identifying the top 10 hub genes based on 12 algorithms

Rank	MCC	DMNC	MNC	Degree	EPC	BottleNeck	EcCentricity	Closeness	Radiality	Betweenness	Stress	ClusteringCoefficient
1	AKT1	RELA	AKT1	AKT1	AKT1	JUN	GSK3B	AKT1	AKT1	AKT1	AKT1	COL18A1
2	PTGS2	CASP1	NFKB1	NFKB1	JUN	PTGS2	CYP3A4	NFKB1	NFKB1	NFKB1	NFKB1	CDK6
3	NFKB1	GSK3B	EGFR	EGFR	NFKB1	PPARG	CDK6	EGFR	EGFR	PPARG	PPARG	TLR9
4	JUN	TLR9	PTGS2	PTGS2	PTGS2	CXCL8	TLR9	PTGS2	PTGS2	EGFR	EGFR	NOS3
5	CXCL8	NOS3	PPARG	PPARG	EGFR	GSK3B	EGFR	PPARG	PPARG	CXCL8	CXCL8	RELA
6	EGFR	PTGS2	JUN	JUN	PPARG	CYP3A4	PTGS2	JUN	JUN	JUN	JUN	CASP1
7	RELA	JUN	CXCL8	CXCL8	CXCL8	COL18A1	PPARG	CXCL8	CXCL8	PTGS2	PTGS2	GSK3B
8	CASP1	CXCL8	GSK3B	GSK3B	GSK3B	CDK6	AKT1	GSK3B	GSK3B	CYP3A4	CYP3A4	PTGS2
9	PPARG	EGFR	RELA	RELA	CASP1	CYP27B1	JUN	RELA	RELA	GSK3B	GSK3B	JUN
10	GSK3B	PPARG	CASP1	CASP1	RELA	TLR9	NFKB1	CASP1	CASP1	RELA	RELA	CXCL8

*MCC* Maximal Clique Centrality, *DMNC* Density of Maximum Neighborhood Component, *MNC* Maximum Neighborhood Component

**Table 2:** Identification of hub genes based on degree value

No.	Target	Gene name	Degree	Uniprot ID	ChEMBL ID	Target Class
1	Serine/threonine-protein kinase AKT	AKT1	14	P31749	CHEMBL4282	Kinase
2	Nuclear factor NF-kappa-B p105 subunit	NFKB1	13	P19838	CHEMBL3251	Other cytosolic protein
3	Epidermal growth factor receptor erbB1	EGFR	12	P00533	CHEMBL203	Kinase
4	Cyclooxygenase-2	PTGS2	12	P35354	CHEMBL230	Oxidoreductase
5	Peroxisome proliferator-activated receptor gamma	PPARG	12	P37231	CHEMBL235	Nuclear receptor

### Results of enrichment analysis

**Fig. 5:**
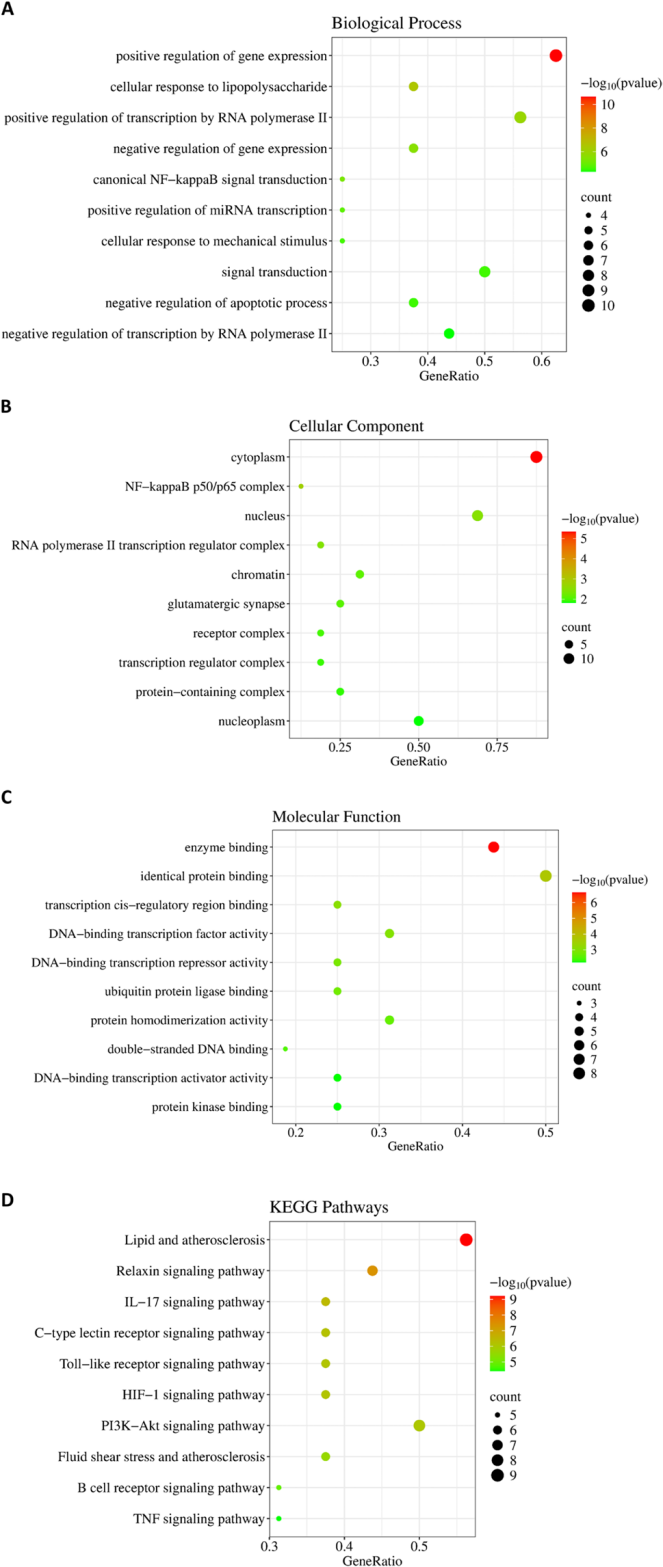
GO functional analysis and KEGG pathway analysis. (**A**) Biological process; (**B**) Cellular component; (**C**) Molecular function; (**D**) KEGG pathway analysis

**Fig. 6:**
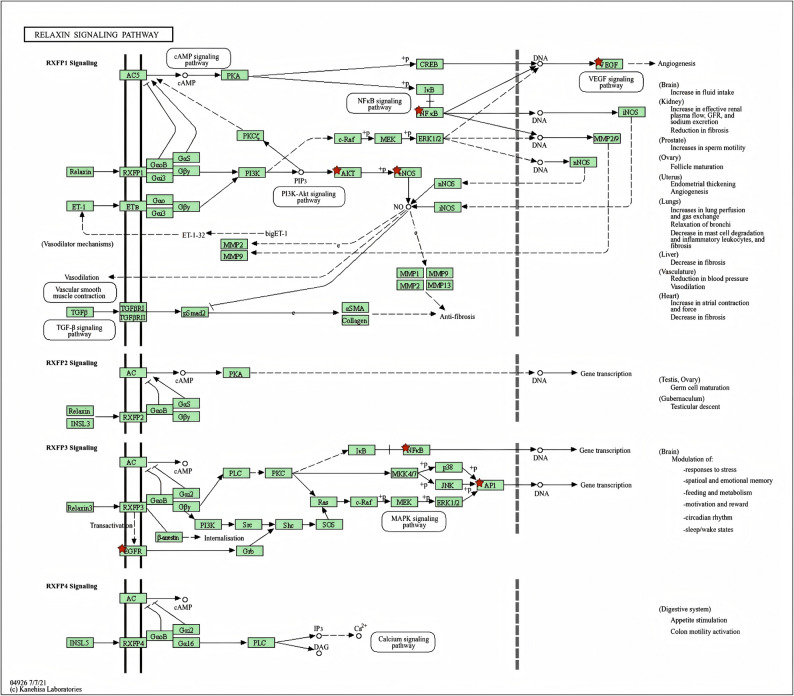
The targets related to the relaxin signaling pathway

By submitting 16 overlapping targets to the David database, enrichment analysis identified a total of 123 biological processes, 14 cellular components, 31 molecular functions, and 103 signaling pathways. Bubble charts were used to illustrate the top 10 enriched items (Fig. 5). Through identical protein binding and enzyme binding, these targets were pivotal in essential biological processes, including regulation of gene expression, regulation of transcription by RNA polymerase II, and signal transduction, which predominantly occurs in the cytoplasm. The relevant signaling pathways of these targets encompassed lipid and atherosclerosis, relaxin signaling pathway, IL-17 signaling pathway, C-type lectin receptor signaling pathway, Toll-like receptor signaling pathway, HIF-1 signaling pathway, PI3K-AKT signaling pathway, fluid shear stress and atherosclerosis, B cell receptor signaling pathway, and TNF signaling pathway. The critical targets related to the relaxin signaling pathway predominantly included JUN, NOS3, AKT1, RELA, EGFR, NFKB1, and VEGFA (Fig. 6). These results suggested that critical targets predominantly function within the cytoplasm through identical protein binding via the relaxin signaling pathway, consequently regulating gene expression, which constitutes the foundational basis for the role of gut microbiota metabolites against VGR. It is important to note that these enrichment results are contingent upon the coverage and annotation biases of the underlying source databases.

### Analysis of the gut microbiota-metabolites-targets-signaling pathways network

**Fig. 7:**
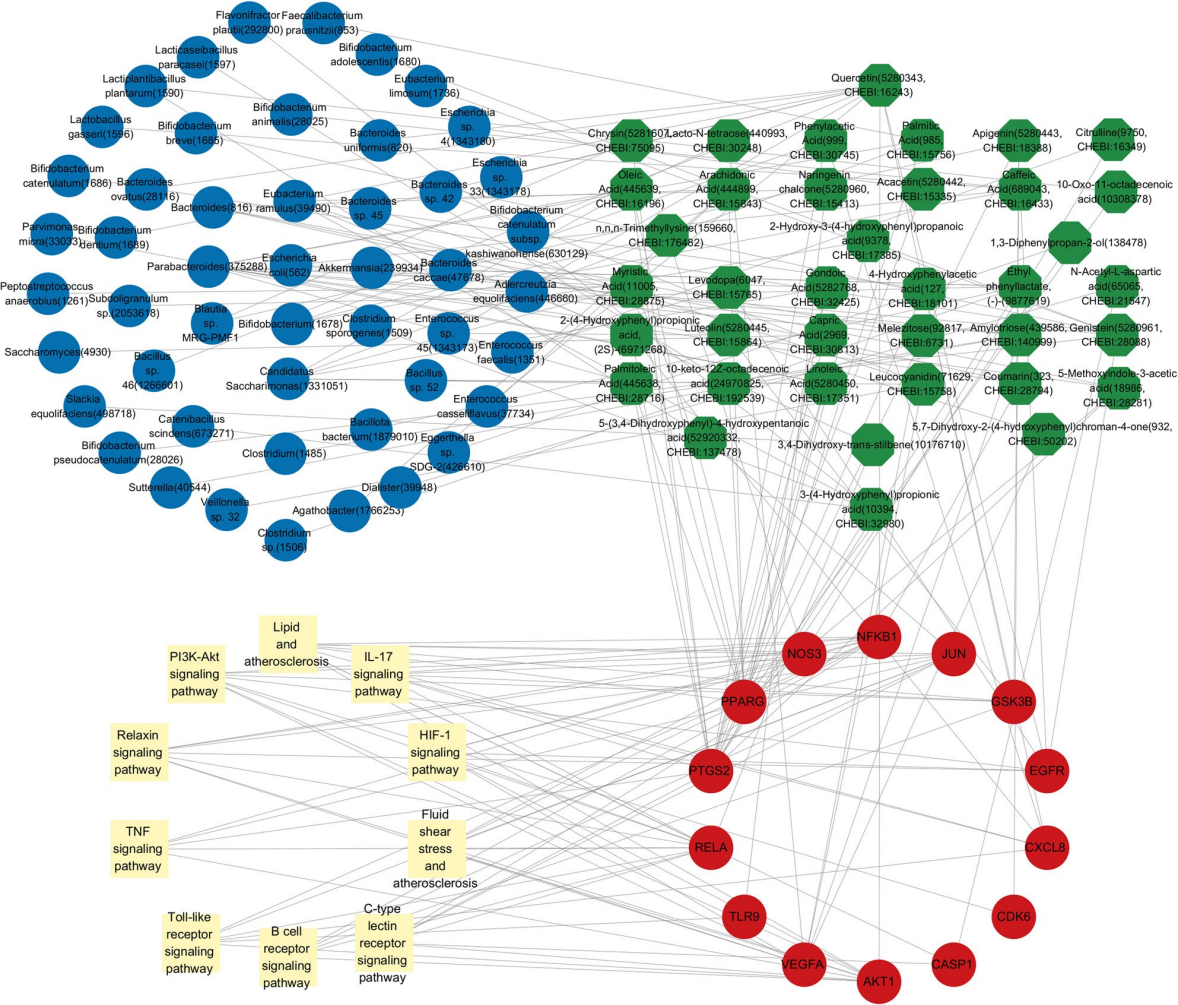
The network of gut microbiota-metabolites-targets-signaling pathways (GM-M-T-SP). In this network, the gut microbiota were depicted in blue, metabolites were represented in green, targets were illustrated in red, and signal pathways were highlighted in yellow

We constructed a gut microbiota-metabolites-targets-signaling pathways (GM-M-T-SP) network, which integrated 111 nodes (49 gut microbiota, 38 metabolites, 14 targets, 10 pathways) and 196 edges (Fig. 7). VDR and CYP3A4 were not incorporated due to their absence of connections to the top 10 signaling pathways. Degree value served as the criterion for screening core metabolites. Table 3 presents these core metabolites, including quercetin, which demonstrate key topological roles in the network. Based on the GM-M-T-SP network architecture, the substitution of key metabolites enables both backward tracing to their putative gut microbial sources and forward mapping to their potential targets and corresponding signaling pathways, thereby elucidating multi-level mechanistic interactions.

**Table 3:** Core metabolites screened by degree

	Compound CID	CHEBI ID	Degree
Quercetin	5,280,343	16,243	11
Genistein	5,280,961	28,088	7
Ethyl phenyllactate, (-)-	9,877,619	-	6
Amylotriose	439,586	140,999	5
Leucocyanidin	71,629	15,758	5

**Fig. 8:**
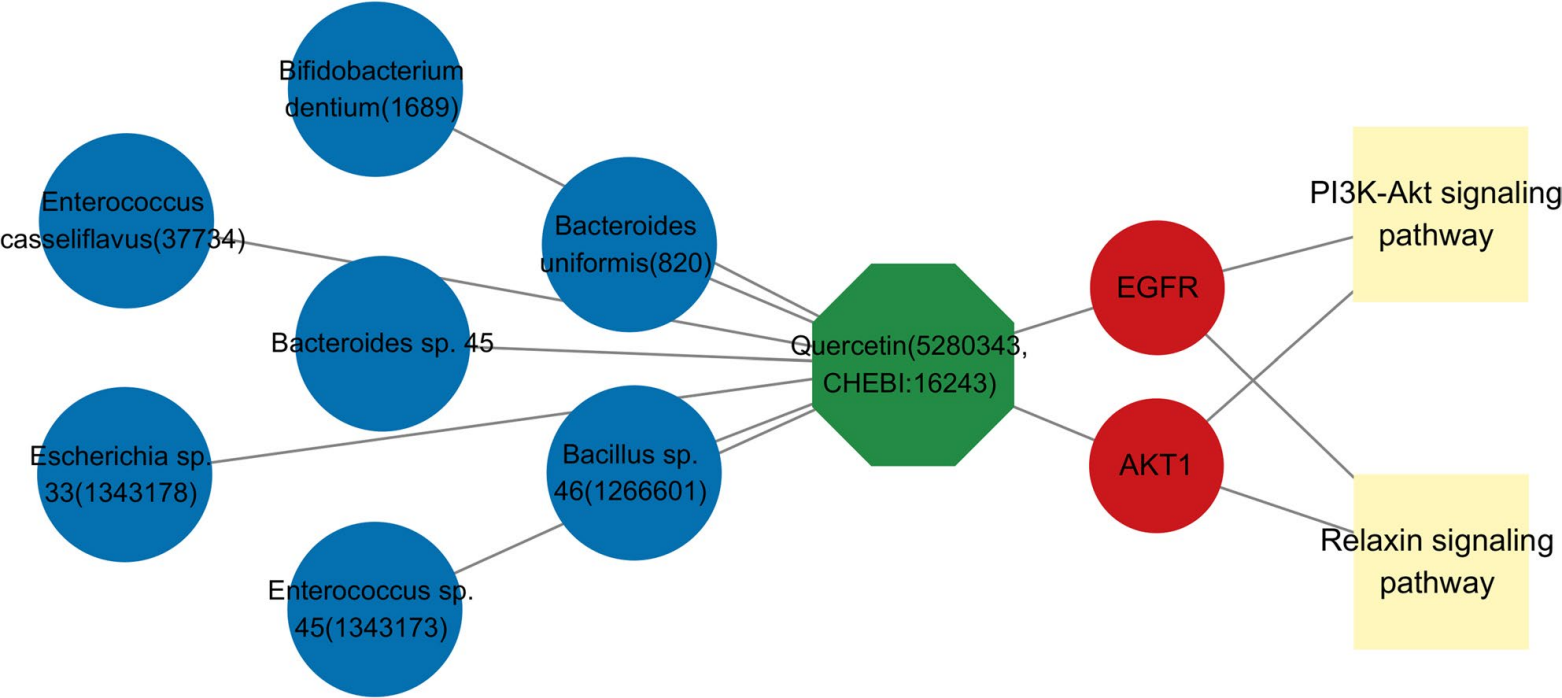
The network of gut microbiota-quercetin-targets-critical signaling pathways (GM-Q-T-P). In this network, the gut microbiota were depicted in blue, quercetin was represented in green, hub targets were illustrated in red, and critical signal pathways were highlighted in yellow

Subsequently, we established a gut microbiota-quercetin-hub targets-critical signaling pathways (GM-Q-T-P) network, which comprises 7 gut microbiota, 1 metabolites, 2 targets, and 2 signaling pathways (Fig. 8). The network topology indicated two database-predicted associations: first, a putative link between quercetin and specific gut microbiota (*Bacillus sp. 46*,* Bacteroides sp. 45*,* Bacteroides uniformis*,* Bifidobacterium dentium*,* Enterococcus casseliflavus*,* Enterococcus sp. 45*,* and Escherichia sp. 33*); and second, a putative central mechanism whereby quercetin may interact with AKT1 and EGFR in the PI3K-AKT signaling pathway.

### Drug-likeness and toxicity assessment

To investigate pharmacological properties and potential harmful impacts, we conducted drug-likeness and toxicity assessments on the top 5 metabolites identified in the network. These metabolites were analyzed using the SwissADME database, which revealed that quercetin, genistein, and ethyl phenyllactate (-)- fully adhere to Lipinski’s rule of five (Table 4).

**Table 4:** Evaluation of drug-likeness using SwissADME

	Compound CID	Molecular weight (g/mol)	H-bond acceptors	H-bond donors	Consensus Log Po/w	TPSA (Å²)	Bioavailability Score	Lipinski’s Rule of Five
Quercetin	5,280,343	302.24	7	5	1.23	131.36	0.55	Yes
Genistein	5,280,961	270.24	5	3	2.04	90.9	0.55	Yes
Ethyl phenyllactate, (-)-	9,877,619	194.23	3	1	1.81	46.53	0.55	Yes
Amylotriose	439,586	504.44	16	11	−5.44	268.68	0.17	No
Leucocyanidin	71,629	306.27	7	6	0.07	130.61	0.55	No

Subsequently, the ADMETlab 2.0 database was utilized to evaluate drug toxicity for these metabolites (Table 5). Preliminary in silico prediction using ADMETlab 2.0 indicated potential hepatotoxicity and genotoxicity risks associated with quercetin. However, given the inherent limitations of computational models in predicting the toxicity of natural compounds, we conducted an extensive data review on metabolites. According to records from the PubChem database and the LiverTox database, quercetin has not been associated with significant liver toxicity or carcinogenic risk related to genotoxicity in human studies. Thus, despite the initial alert raised by the predictive model, both animal and clinical data support the safety of quercetin at appropriate dosages. In contrast, PubChem data indicated that high-dose genistein (500 ppm) exhibits certain carcinogenic effects in animal models. Furthermore, no safety evidence regarding ethyl phenyllactate (-)- was available in either the PubChem or the LiverTox database.

**Table 5:** Evaluation of toxicity using ADMETlab 2.0

	Compound CID	hERG Blockers	H-HT	DILI	AMES Toxicity	Rat Oral Acute Toxicity	FDAMDD	Carcinogencity	Respiratory Toxicity
Quercetin	5,280,343	---	---	+++	+	---	-	---	---
Genistein	5,280,961	---	---	+	--	-	--	-	---
Ethyl phenyllactate, (-)-	9,877,619	---	---	--	---	---	-	---	-
Amylotriose	439,586	-	---	--	---	---	---	---	---
Leucocyanidin	71,629	---	---	--	+	--	---	---	---

The favorable drug-likeness and safety predicted for quercetin, genistein, and ethyl phenyllactate (-)- by multi-database analysis must be viewed in light of a fundamental limitation: such tools are inherently constrained by existing data on known compounds and may miss uncommon adverse effects. Therefore, despite the promising potential of quercetin suggested by network pharmacology, the computational flagging of DILI risk mandates careful dosage monitoring in any future preclinical and clinical evaluation.

### Evaluation of the docking protocol

Prior to analyzing the binding mode of quercetin, the accuracy of the molecular docking protocol was assessed. Re-docking procedures were performed for targets containing co-crystallized ligands: AKT1 (1UNQ), NFKB1 (8TQD), EGFR (8A27), and PTGS2 (5F19). The results showed that the re-docked conformations of the native ligands closely superimposed with their original crystallographic poses. The calculated RMSD values were 1.404 Å for AKT1, 0.736 Å for NFKB1, 1.096 Å for EGFR, and 0 Å for PTGS2. All values are well below the accepted threshold of 2.0 Å, confirming that the docking parameters and strategy employed in this study are capable of accurately reproducing the experimentally observed binding modes, thereby ensuring the reliability of the subsequent docking results.

### Molecular docking results of quercetin and positive control ligands

To investigate the multi-target potential of quercetin, molecular docking was performed against the active sites of AKT1, NFKB1, EGFR, PTGS2, and PPARG. The results demonstrated that quercetin could stably bind to all five targets, with binding affinities ranging from − 6.0 to −8.9 kcal/mol, suggesting a favorable binding tendency. For reference, the positive control ligands could bind to their respective targets, with binding affinities ranging from − 5.3 to −8.8 kcal/mol. The binding energy of quercetin for AKT1, NFKB1, EGFR, and PPARG was comparable to or more favorable than that of the known positive controls, whereas its affinity for PTGS2 was less favorable. Table 6 summarizes these comparative results.

**Table 6:** Computational molecular Docking results summary

Protein target (PDB ID)	Chemical component (PubChem CID)	Binding energy (kcal/mol)
AKT1 (1UNQ)	Quercetin (5280343)	−6.0
AKT1 (1UNQ)	Ipatasertib (24788740)	−5.3
NFKB1 (8TQD)	Quercetin (5280343)	−6.9
NFKB1 (8TQD)	Parthenolide (7251185)	−6.2
EGFR (8A27)	Quercetin (5280343)	−8.9
EGFR (8A27)	Gefitinib (123631)	−8.8
PTGS2 (5F19)	Quercetin (5280343)	−7.4
PTGS2 (5F19)	Celecoxib (2662)	−8.8
PPARG (8BF1)	Quercetin (5280343)	−7.7
PPARG (8BF1)	Rosiglitazone (77999)	−7.4

**Fig. 9:**
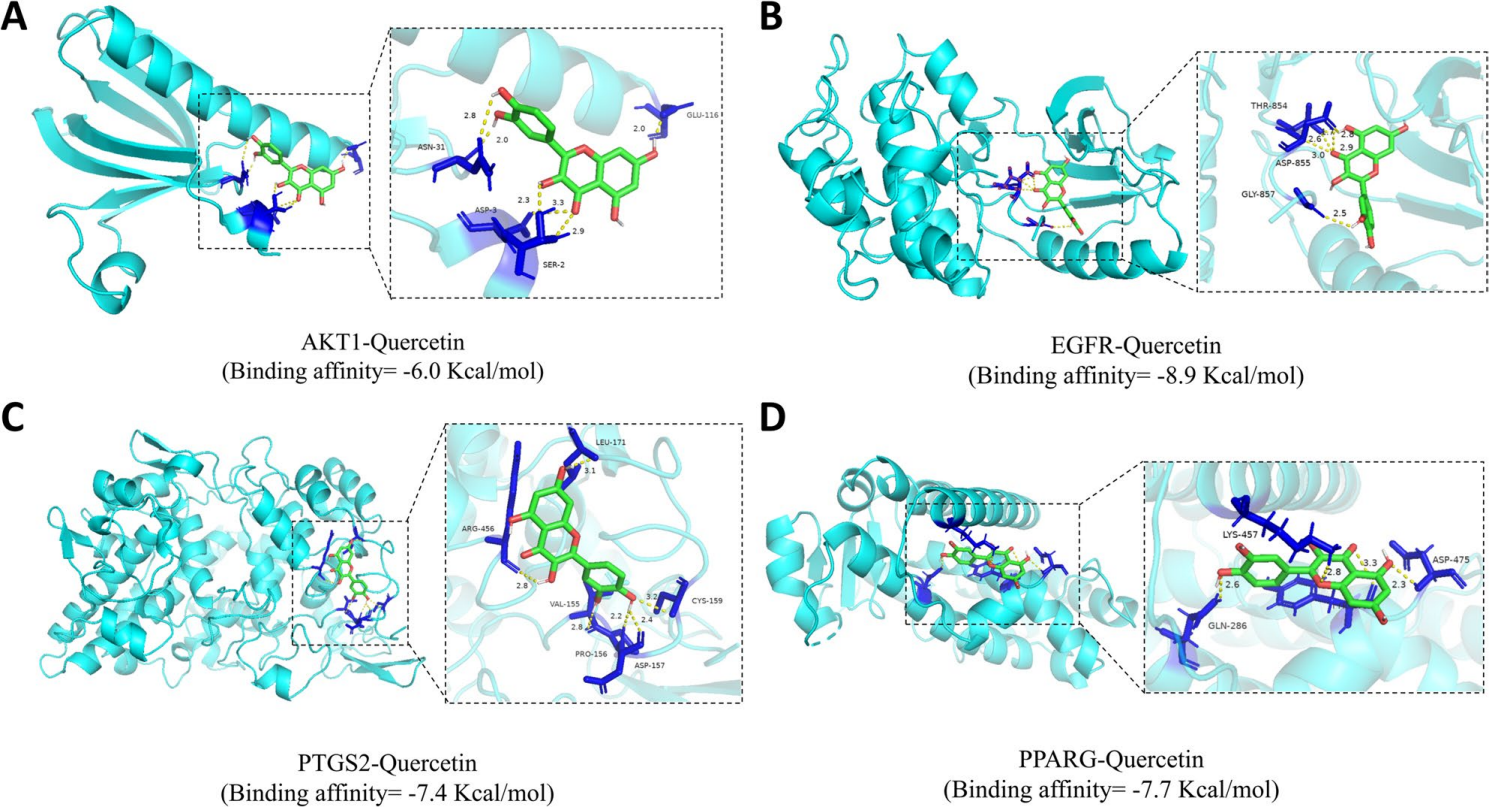
The binding modes of hub targets and quercetin. (**A**) AKT1-Quercetin; (**B**) EGFR-Quercetin; (**C**) PTGS2-Quercetin; (**D**) PPARG-Quercetin

Specifically, quercetin formed the most stable complex with EGFR, exhibiting a binding energy of −8.9 kcal/mol. The interaction was primarily mediated by a critical hydrogen bond with Thr854, a π-cation interaction with Lys745, and hydrophobic interactions with Ala743, Ile744, Met766, Ala763, and Ile759. For the nuclear receptor PPARG, quercetin was successfully docked into its ligand-binding domain with a binding energy of −7.7 kcal/mol. Interaction analysis indicated that quercetin formed a key hydrogen bond with Asp475, a π-π stacking interaction with Tyr473, and a π-cation interaction with Lys457, while also engaging in extensive hydrophobic contacts with Leu465, Met463, and Val450. Notably, within the active site of PTGS2, quercetin established a critical hydrogen bond with Pro156, supplemented by hydrophobic interactions with Val155, Cys159, Pro162, and Leu171, with a binding energy of −7.4 kcal/mol. In contrast, the interaction with NFKB1 was distinct, characterized by strong polar interactions with catalytic site residues Thr145 and His143, resulting in a binding energy of −6.9 kcal/mol. Finally, the complex between quercetin and AKT1 exhibited a binding energy of −6.0 kcal/mol, characterized by hydrogen bonds to Glu116 and Asn31 and accompanied by hydrophobic contacts with Leu110 and Val106. Based on the molecular docking results, quercetin was predicted to form stable conformations within the binding pockets of hub targets. These in silico analyses propose putative hydrogen bond interactions between quercetin and AKT1, EGFR, PTGS2, and PPARG (Fig. 9), although this requires validation through experimental studies.

### Analysis of molecular dynamics simulations

**Fig. 10:**
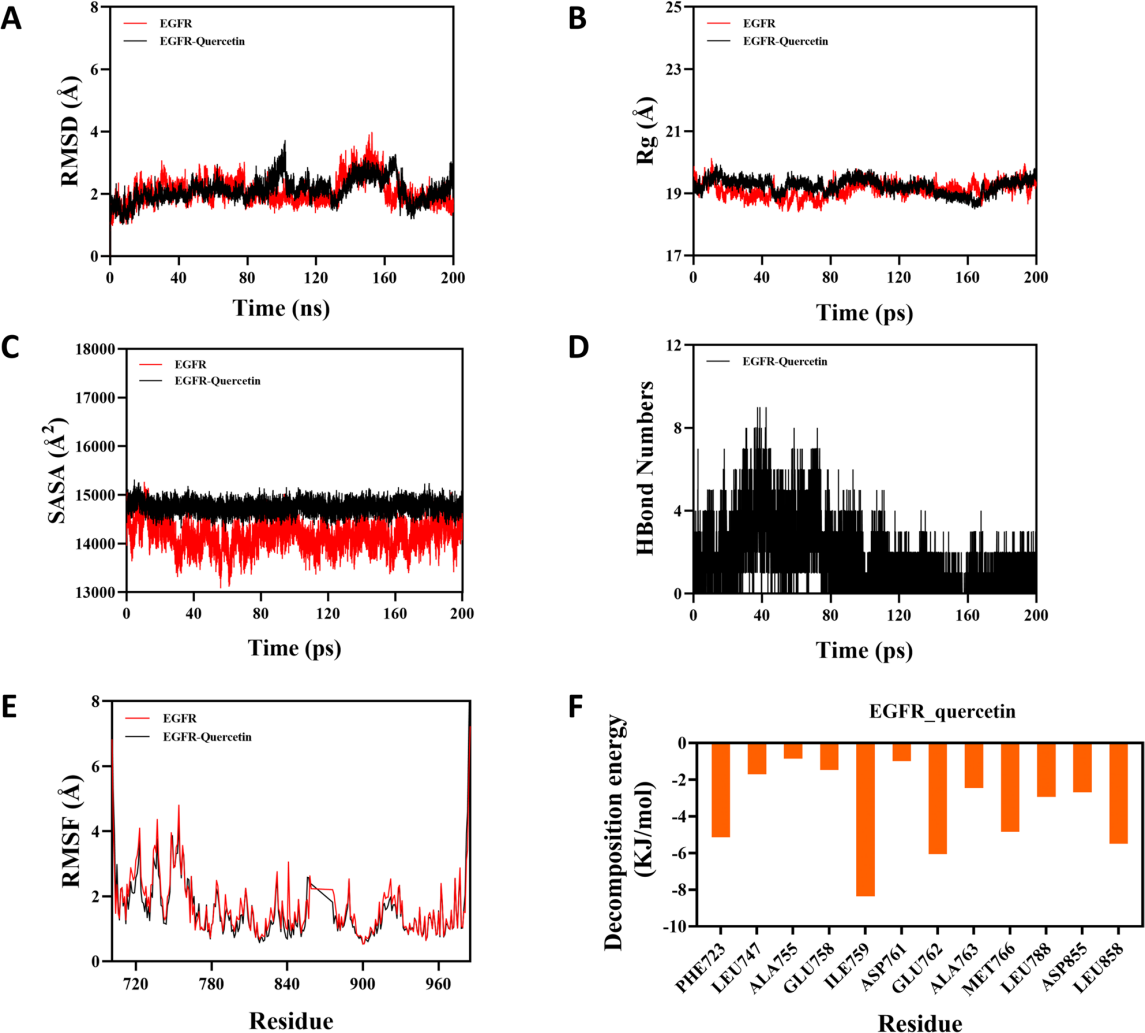
Results of molecular dynamics simulations. (**A**) Root mean square deviation (RMSD). (**B**) Radius of gyration (Rg). (**C**) Solvent-accessible surface area (SASA). (**D**) Quantity of hydrogen bonds. (**E**) Root mean square fluctuation (RMSF). (**F**) Decomposition energy of EGFR-Quercetin complex system

To complement the molecular docking results, molecular dynamics simulations were performed to assess the binding stability and dynamic behavior of the EGFR–quercetin complex. Following equilibration periods (160 ns for EGFR and 180 ns for the EGFR–quercetin complex), RMSD analysis showed that both systems attained stable states with fluctuations around 1.6 Å and 2.4 Å, respectively (Fig. 10A). The comparable stability profiles suggest that quercetin binding contributes to the overall stability of the protein-ligand system. Computational data indicated that the Rg for both the EGFR protein and the EGFR-quercetin complex underwent only minor fluctuations (Fig. 10B). This could be interpreted as the system maintaining a relatively stable compactness during the simulated interaction. Analysis of the SASA for the EGFR-quercetin complex upon ligand binding showed a relatively minor change, suggesting that ligand binding induces minimal perturbation to the protein’s structure (Fig. 10C). Hydrogen bond analysis revealed that the EGFR–quercetin complex maintained between 0 and 9 hydrogen bonds, with an average of 3 bonds present during the simulation (Fig. 10D). Finally, the RMSF of residue motions remained largely below 3 Å, indicating reduced flexibility and enhanced structural stability across the protein backbone (Fig. 10E). MM/PBSA binding free energy calculations further quantified the interaction between EGFR and quercetin, with a favorable binding affinity of −118.748 kJ/mol. Binding energy decomposition of identified several key residues, including Ile759, Glu762, and Leu858, Phe723, and Met766, were major contributors to complex stabiization, underscoring their critical roles in the binding process (Fig. 10F). These mechanistic insights should be interpreted with caution pending experimental confirmation.

## Discussion

The growing recognition of the gut microbiota’s indispensable role in metabolizing and bioactivating natural flavonoids is driving the concept of metabolite repurposing for cardiovascular diseases into greater focus. Most dietary flavonoids are ingested as glycosides with limited bioavailability and activity until gut microbial enzymes (e.g., glucosidases, rhamnosidases) hydrolyze them into more bioactive aglycones, thereby enabling subsequent microbial transformations [27]. Against this backdrop, the pathogenesis of VGR likely represents a multifactorial process involving complex host-microbiota interactions. Through an integrative bioinformatics strategy that constructed a GM–M–T–V network, we systematically decoded these mechanisms and identified quercetin as a core microbiota-derived metabolite with multi-targeting potential against hub genes. Computational simulations further support its potential therapeutic relevance, demonstrating favorable binding affinity and dynamic stability toward targets involved in critical signaling pathways associated with VGR.

In this study, network pharmacology identified hub targets of metabolites with potential therapeutic significance. Among these, AKT1 serves as a central signaling node that regulates multiple downstream pathways, particularly the PI3K/AKT/mTOR pathway, thereby influencing the proliferation, migration, and survival of endothelial cells, which is fundamental to vascular remodeling [28]. NFKB1, a pivotal transcription factor involved in immune and inflammatory responses, contributes to the chronic inflammation observed in cardiovascular pathogenesis [29]. Its activation promotes the secretion of pro-inflammatory cytokines, which in turn sustains and amplifies local inflammatory cascades, creating a self-perpetuating cycle that fosters vascular remodeling and restenosis [30]. Furthermore, EGFR overexpression has been shown to exacerbate inflammatory responses and potentiate NF-κB signaling via cross-activation of the PI3K/AKT pathway. This signaling interplay enhances the proliferation and migration of VSMCs, thereby driving pathological vascular remodeling [31]. Although these hub genes provide mechanistic insights into the pathogenesis of VGR and highlight potential therapeutic targets, it is important to acknowledge that network pharmacology-based predictions are inherently hypothetical. Limitations such as incomplete mapping of biomolecular interactions, potential false positives in functional enrichment analyses, and dependence on partially curated database annotations underscore the need for further experimental validation.

Enrichment analysis was conducted to elucidate the mechanisms through which gut microbiota metabolites regulate the relaxin signaling pathway. GO analysis indicated that the identified protein targets are predominantly localized in the cytoplasm and engage in identical protein binding and enzyme binding interactions. These molecular functions are integrated through the relaxin signaling pathway to regulate gene expression. Identical protein binding facilitates multi-protein complex assembly, with transcription factors playing a central role in recognizing and binding specific genomic regions to regulate downstream gene expression. Enzyme binding, particularly through kinases and phosphatases, catalyzes post-translational modifications—such as phosphorylation and dephosphorylation—that dynamically regulate transcription factor activity and DNA-binding affinity, thereby fine-tuning gene expression in response to physiological cues [32]. Mechanistically, the relaxin signaling pathway has gained recognition as a key regulator of extracellular matrix homeostasis and fibrotic processes in cardiovascular contexts. Relaxin signaling exerts its anti-fibrotic effects by activating the NO/cGMP pathway, which mediates the suppression of Angiotensin II-induced cardiac fibroblast proliferation, migration, and extracellular matrix remodeling through upregulation of total nitric oxide synthase (TNOS) activity and subsequent restoration of nitric oxide bioavailability [33]. Additionally, relaxin signaling via RXFP1 modulates downstream signaling cascades that drive phenotypic switching and migration in VSMCs [34]. Importantly, crosstalk between the relaxin and PI3K-AKT pathways has been observed, suggesting synergistic regulation of VSMCs proliferation and migration—a mechanism potentially relevant to the attenuation of VGR [35, 36]. Despite establishing the relaxin pathway as a promising target against vascular remodeling, its relative contribution to these intersecting pathways remains unclear, mandating further mechanistic and translational studies.

Through integrated computational prioritization, quercetin was identified as a key gut microbiota-derived metabolite with high network connectivity, favorable drug-likeness, and a low toxicity profile. As a naturally occurring flavonoid, quercetin exhibits multiple cardioprotective effects—including antioxidant, anti-inflammatory, antihypertensive, and lipid-lowering activities [37–39]. In conventionally raised ApoE⁻/⁻ mice, quercetin exerts atheroprotective effects by reducing atherosclerotic lesion size and macrophage accumulation, a process dependent on gut microbiota that metabolize quercetin into phenolic metabolites like 3,4-dihydroxybenzoic acid, suggesting a protective mechanism involving endothelial barrier integrity that is modulated by dietary microbiota-accessible carbohydrates [40]. Beyond its anti-atherogenic properties, quercetin mitigates oxidative stress via inhibition of NADPH oxidase and xanthine oxidase, and suppresses macrophage secretion of pro-inflammatory cytokines including TNF-α, IL−6, and IL−1β [41, 42]. Experimentally, quercetin attenuates myocardial ischemia-reperfusion injury and reduces infarct size [43]. Despite being confined to the problematic free form in clinical trials, quercetin has shown marked therapeutic potential [44]. Structurally, the bioactivity of quercetin is closely linked to specific substituents that influence its solubility, stability, and target engagement [45, 46]. Critically, its efficacy depends on microbial biotransformation: commensal bacteria such as *Bacillus*, *Bacteroides*, and *Bifidobacterium*, express enzymatic systems that hydrolyze glycosylated quercetin into absorbable aglycones and phenolic metabolites, substantially enhancing systemic bioavailability [47–49]. Although quercetin emerges from our analysis as a promising gut microbiota-derived metabolite for inhibiting VGR, several challenges remain. Its clinical translation is limited by inherently low bioavailability, and emerging evidence suggests that microbial derived secondary metabolites of quercetin may drive much of its observed bioactivity. Future studies should therefore focus on clarifying the metabolic cascade and identifying the ultimate active species responsible for its pharmacological effects.

Molecular docking analysis suggested that quercetin adopts a binding mode highly similar to that of the canonical EGFR inhibitor gefitinib, with both ligands engaging residues Lys745 and Ala743 within the ATP-binding pocket [50, 51]. The hydrogen bond interaction with Lys745, a critical anchor point for ATP-competitive inhibitors, supports a shared molecular mechanism of action [52]. This common binding motif is further stabilized by hydrophobic contacts near Ala743, providing a structural basis for the inhibitory activity observed in silico [53]. Expanding on these molecular docking results, MD simulations revealed a more detailed interaction profile and highlighted residues that contribute to the stability and specificity of quercetin binding. Beyond the conserved interactions with Lys745 and Ala743, several additional residues, such as Ile759, Leu858, and Phe723, form a hydrophobic subpocket that enhances complex stability through sustained van der Waals contacts [54, 55]. Notably, interactions with Met766 and Glu762, potentially involving hydrogen bonding or a salt bridge, indicate a distinct binding pattern that may differentiate quercetin from gefitinib [56]. This extended network of interactions, particularly the engagement of the hydrophobic cleft and salt-bridge formation with Glu762, implies not only improved binding affinity but also potential selectivity advantages that could help circumvent mutation-induced resistance in EGFR [57]. While the convergence of molecular docking and MD simulations offers structural insights into quercetin’s mechanism, these computational approaches remain limited in modeling full physiological complexity. Experimental studies are therefore essential to validate these predictions and clarify the functional implications of the observed binding behavior.

This study has several limitations that should be considered when interpreting the findings. The primary limitation of our identification strategy concerns the exclusive use of canonical SMILES for structural representation. Although canonical SMILES offer a standardized 2D molecular framework, they lack stereospecificity and cannot differentiate between critical isomeric forms, which are defined in isomeric SMILES descriptors. This restricts our workflow from identifying distinct stereoisomers with potentially unique biological roles. An additional limitation of this study lies in the fact that the entire analysis relies on computational predictions and public genomic data. While our integrated approach identified quercetin as a key metabolite and linked it to the relaxin signaling pathway, these associations remain hypothetical without experimental validation. The absence of in vivo and in vitro experiments remains a notable limitation, precluding definitive conclusions regarding the proposed mechanisms.It should be noted that the reliability of our network pharmacology results is contingent upon the completeness and accuracy of the underlying databases. Potential biases in these datasets could affect the outcome. Consequently, the proposed axis from gut microbiota to VGR, though biologically plausible, is largely speculative and requires direct evidence to establish causality. Future research should incorporate complementary in vivo and in vitro experiments to validate our findings.

## Conclusion

This study suggests that the inhibition of VGR by gut microbiota metabolites may involve interactions with AKT1, NFKB1, EGFR, PTGS2 and PPARG, as well as participation in the relaxin signaling pathway. Furthermore, quercetin is identified as a promising candidate metabolite for therapeutic development. Collectively, these findings derived from integrated computational and database analyses lay the groundwork for novel early intervention strategies against VGR and underscore the necessity for subsequent experimental verification.

## Supplementary Information

Supplementary Material 1: Additional fle 1: Table S1: Associations between gut microbiota and metabolites

Supplementary Material 2: Additional fle 2: Table S2: Inventory of metabolites and gut microbiota from reverse screening

## Data Availability

No datasets were generated or analysed during the current study.

## Abbreviations

CABG: Coronary Artery Bypass Grafting
CHD: Coronary Heart Disease
VGR: Vein Graft Restenosis
VSMCs: Vascular Smooth Muscle Cells
SCFAs: Short-chain Fatty Acids
NP: Network Pharmacology
OMIM: Online Mendelian Inheritance in Man
PPI: Protein-protein Interaction
MDM: Molecular Docking Method
MD: Molecular Dynamics
GO: Gene Ontology
BP: Biological Process
CC: Cellular Component
MF: Molecular Function
KEGG: Kyoto Encyclopedia of Genes and Genomes
AKT1: V-Akt murine thymoma viral oncogene homolog 1
NFKB1: Nuclear factor kappa B subunit 1
EGFR: Epidermal Growth Factor Receptor
PTGS2: Prostaglandin-endoperoxide synthase 2
PPARG: Peroxisome proliferative activated receptor, gamma
RELA: V-Rel reticuloendotheliosis viral oncogene homolog A
VEGFA: Vascular endothelial growth factor A
VDR: Vitamin D receptor
CYP3A4: Cytochrome P450, family 3, subfamily A, polypeptide 43
GSK3B: Glycogen Synthase Kinase 3 Beta
NOS3: Nitric Oxide Synthase 3
CXCL8: C-X-C motif chemokine ligand 8
CDK6: Cyclin-dependent kinase 6
CASP1: Caspase 1
TLR9: Toll-like receptor 9
JUN: Jun proto-oncogene
